# A Good Patron to the Dentist

**Published:** 1854-04

**Authors:** 


*A good Patron to the Dentist.*—A paragraph has been going the rounds in the papers, stating that the heirs of a Parisian dentist had recently brought suit for the recovery of about four thousand dollars, for twelve sets of artificial teeth furnished from 1841 to 1852, to a countess, famous, at the restoration, for her wit and beauty. Fifteen or twenty *paying* patrons of this sort would constitute quite a desirable acquisition to the practice of most dentists, but we would be decidedly opposed to so long a running account.

